# Substitution rate variation at human CpG sites correlates with non-CpG divergence, methylation level and GC content

**DOI:** 10.1186/gb-2011-12-6-r58

**Published:** 2011-06-22

**Authors:** Carina F Mugal, Hans Ellegren

**Affiliations:** 1 Department of Evolutionary Biology, Uppsala University, Norbyvägen 18D, SE-752 36, Uppsala, Sweden

## Abstract

**Background:**

A major goal in the study of molecular evolution is to unravel the mechanisms that induce variation in the germ line mutation rate and in the genome-wide mutation profile. The rate of germ line mutation is considerably higher for cytosines at CpG sites than for any other nucleotide in the human genome, an increase commonly attributed to cytosine methylation at CpG sites. The CpG mutation rate, however, is not uniform across the genome and, as methylation levels have recently been shown to vary throughout the genome, it has been hypothesized that methylation status may govern variation in the rate of CpG mutation.

**Results:**

Here, we use genome-wide methylation data from human sperm cells to investigate the impact of DNA methylation on the CpG substitution rate in introns of human genes. We find that there is a significant correlation between the extent of methylation and the substitution rate at CpG sites. Further, we show that the CpG substitution rate is positively correlated with non-CpG divergence, suggesting susceptibility to factors responsible for the general mutation rate in the genome, and negatively correlated with GC content. We only observe a minor contribution of gene expression level, while recombination rate appears to have no significant effect.

**Conclusions:**

Our study provides the first direct empirical support for the hypothesis that variation in the level of germ line methylation contributes to substitution rate variation at CpG sites. Moreover, we show that other genomic features also impact on CpG substitution rate variation.

## Background

The rate of germ line mutation is the ultimate parameter governing the amount of genetic diversity within populations and the divergence between species. There is extensive variation in mutation rate within genomes and a number of genomic features have been shown to correlate with this rate variation, both at the whole-chromosome level and at regional as well as local levels [1-5]. Examples of factors suggested to be related to mutation rate variation are genetic recombination [6,7], transcription [8,9], replication [10,11], chromatin structure [12,13], distance to telomeres [5], exon density [14] and sequence variables, such as the local GC content [14,15]. Several of these factors are strongly interrelated with each other, which complicates unraveling the driving forces of mutation rate variation.

Sequence context effects modulate the mutation rate at individual nucleotide sites [16]. The most well-known and strongest of these effects is the about one order of magnitude higher frequency of C to T substitutions in CpG dinucleotides than that of other transitions in the genome [2,17-19]. The elevated mutability of cytosines at CpG sites is due to the strong tendency of cytosine residues in CpG dinucleotides to be methylated (^m^CpG) and the associated tendency for cytosine to be replaced by thymine [20,21]. This process is initiated via a spontaneous deamination reaction of 5-methylcytosine directly leading to thymine, which is less efficiently repaired by the DNA repair machinery than the cytosine to uracil deamination reaction [20].

It has recently become clear that the degree of DNA methylation varies significantly throughout the genome, an insight reached by the ability to perform whole-genome analysis of DNA methylation status using massive parallel sequencing coupled with bisulfite-treated DNA or immunoprecipitated methylated DNA [22-24]. The availability of whole-genome methylation maps provides an opportunity to integrate DNA methylation level in models explaining the variation in CpG mutability across the genome, which allows refinement of studies about CpG mutation rate variation. Specifically, rather than considering CpG sites as having a uniformly high rate of mutation due to methylation, it may be that there is variation in this high rate due to variation in methylation level.

Here we made use of whole-genome methylation data from human sperm cells to quantify the contribution of the level of cytosine methylation to germ line CpG mutability in intronic regions of the human genome. In order to control for factors that are not specifically affecting CpG sites, we incorporated non-CpG divergence in our analysis. We then investigated the correlation between CpG substitution rate variation and DNA methylation level, intronic GC content, germ line transcription level and recombination rate. We find a positive correlation of CpG substitution rate with DNA methylation level and non-CpG divergence, and a negative correlation with GC content.

## Results

We estimated human-specific CpG transition and transversion rate, and CpH transition rate, using alignments of orthologous human, rhesus macaque and mouse sequences for a set of 56,363 introns. For downstream analysis we restricted the data to a set of 38,586 introns for which estimates of five possible explanatory variables (non-CpG divergence, DNA methylation level, intronic GC content, germ line transcription level and recombination rate) were available. To investigate differences of the five explanatory variables among the three datasets defined by the presence or absence of CpG islands (CGIs) and DNase I hypersensitive sites (DHSs) (see Materials and methods), we computed their mean values and variances for each dataset (Table 1). As expected for regions containing CGIs, set C has lower methylation level, higher GC content and a higher ratio of observed versus expected CpG content (CpG_[o/e]_). In light of the expected impact of selection on regions containing CGIs and DHSs, non-CpG divergence is unexpectedly similar for the three datasets.

**Table 1:** Mean values and variances of the explanatory variables for datasets A, B and C

	Dataset A		Dataset B		Dataset C	
			
	Mean	Variance	Mean	Variance	Mean	Variance
Non-CpG divergence	0.0206	3.98e-05	0.0207	3.00e-05	0.0203	2.93e-05
Methylation level	0.7723	1.17e-02	0.7588	1.45e-02	0.4348	4.09e-02
GC content	0.3961	3.28e-03	0.4221	3.93e-03	0.4719	5.42e-03
Transcription level	2.5920	4.22e-01	2.5770	4.00e-01	2.6240	3.98e-01
Female recombination rate^a^	0.0721	2.03e-01	0.0832	2.23e-01	0.0462	3.08e-01
Male recombination rate^a^	-0.3969	5.01e-01	-0.3380	4.50e-01	-0.3551	5.27e-01
CpG[o/e]^b^	0.4168	1.85e-02	0.4334	1.68e-02	0.7026	6.82e-02

Dataset A contains no CpG islands (CGIs) or DNase I hypersensitive sites (DHSs)), dataset B contains DHSs but no CGIs, and dataset C contains both CGIs and DHSs. ^a^Log-transformed values. ^b^Observed versus expected CpG content.

We performed logit-regression analysis using CpG transition rate as response variable. Variance inflation factors were all < 2, so multi-colinearity was only a minor issue. The regression analyses showed that, for all three datasets, variation in CpG transition rate was mainly explained by variation in non-CpG divergence, DNA methylation level and GC content. It was positively correlated with divergence and methylation level and negatively correlated with GC content. Germ line transcription level was of subordinate importance, while the impact of female and male recombination rate was negligible. Estimates of the standardized slopes and their significance levels are listed in Table 2. While for datasets A and B non-CpG divergence was the dominant explanatory variable (standardized slopes of 0.1832 and 0.1574, respectively, compared to -0.1036 and -0.1382 for GC content and 0.0385 and 0.1047 for DNA methylation level), DNA methylation level showed the strongest impact for dataset C (0.3454 compared to -0.1849 for GC content and 0.1722 for non-CpG divergence). The percentage of explained deviance was 17.4, 33.6 and 70.0 for datasets A, B and C, respectively.

**Table 2:** Summary of parameter estimates and significance levels of the multivariate generalized linear regression analysis for CpG transition rate

	Dataset A		Dataset B		Dataset C	
			
	Estimate	-log_10_(p)	Estimate	-log_10_(p)	Estimate	-log_10_(p)
Divergence	0.1832	> 15.70	0.1574	> 15.70	0.1722	> 15.70
Methylation level	0.0385	9.50	0.1047	> 15.70	0.3454	> 15.70
GC content	-0.1036	> 15.70	-0.1382	> 15.70	-0.1849	> 15.70
Transcription level	-0.0145	1.95	-0.0094	3.01	-0.0114	1.46
Female recombination rate	0.0031	0.22	0	0	0.0158	2.29
Male recombination rate	-0.0054	0.43	-0.0084	2.29	-0.0167	2.42
Explained deviance	1,243		5,310		8,038	
Residual deviance	5,894		10,496		3,451	
n^a^	13,038		21,636		3,871	
AIC^b^	37,300		78,030		17,172	

Dataset A contains no CpG islands (CGIs) or DNase I hypersensitive sites (DHSs)), dataset B contains DHSs but no CGIs, and dataset C contains both CGIs and DHSs. ^a^Sample size. ^b^Akaike Information Criterion.

To assess the strand bias in CpG transition rate, we computed the difference between CpG transition rate on the coding and on the non-coding strand. We found 95% confidence intervals (-0.275, 0.237) for dataset A, (-0.204, 0.174) for dataset B and (-0.096, 0.085) for dataset C. Thus, as differences in rates were distributed around zero, we could not find evidence for transcription-induced strand bias in CpG transition rate in any of the three datasets.

We repeated the above analysis using CpG transversion rate as the response variable. Estimates of the standardized slopes and their significance levels are listed in Table 3.

**Table 3:** Summary of parameter estimates and significance levels of the multivariate generalized linear regression analysis for CpG transversion rate

	Dataset A		Dataset B		Dataset C	
			
	Estimate	-log_10_(p)	Estimate	-log_10_(p)	Estimate	-log_10_(p)
Divergence	0.2044	> 15.70	0.1823	> 15.70	0.1414	> 15.70
Methylation level	0.0222	1.14	0.0534	14.88	0.0781	> 15.70
GC content	-0.0918	> 15.70	-0.1401	> 15.70	-0.1076	> 15.70
Transcription level	-0.0440	3.84	-0.0322	8.15	-0.0336	3.64
Female recombination rate	0.0071	0.26	-0.0084	0.83	0.0044	0.19
Male recombination rate	0.0022	0.07	-0.0006	0.04	-0.0011	0.04
Explained deviance	356		1,421		519	
Residual deviance	9,979		15,544		2,986	
n^a^	13,038		21,636		3,871	
AIC^b^	21,460		50,718		12,684	

Dataset A contains no CpG islands (CGIs) or DNase I hypersensitive sites (DHSs)), dataset B contains DHSs but no CGIs, and dataset C contains both CGIs and DHSs. ^a^Sample size. ^b^Akaike Information Criterion.

For all three datasets non-CpG divergence was the dominating explanatory variable (standardized slopes of 0.2044, 0.1823 and 0.1414 for datasets A, B and C, respectively), followed by GC content (-0.0918, -0.1401 and -0.1076). Transcription level and methylation level were of minor importance (standardized slopes generally <0.05). As for the CpG transition rate, female and male recombination rate were of negligible importance. Notably, the percentage of explained deviance was generally rather low, with values of 3.4, 8.4 and 14.8 for datasets A, B and C, respectively.

In order to investigate the contrasting effects of methylation level on CpG transition and transversion rates in more detail, we analyzed the relationship between the five explanatory variables and the transition/transversion rate ratio (κ) for cytosines located in CpG sites (excluding introns with CpG transversion rate values of zero). We then performed a linear regression analysis for each of the three datasets. Estimates of the standardized slopes and their significance levels are listed in Table 4. For datasets A and B the overall amount of explained variance was low (3.97% and 1.73%, respectively) and there was no clear pattern common to both datasets. However, for dataset C we found the amount of explained variance to be as high as 17.73%. With a standardized slope of 0.8127, methylation level was clearly the dominating explanatory variable, followed by non-CpG divergence (0.2093) and GC content (-0.1815). Germ-line transcription level and female and male recombination rate were of minor importance.

**Table 4:** Summary of parameter estimates and significance levels of the multivariate linear regression analysis for the CpG transition/transversion rate ratio (κ)

	Dataset A		Dataset B		Dataset C	
			
	Estimate	-log_10_(p)	Estimate	-log_10_(p)	Estimate	-log_10_(p)
Divergence	0.2574	15.20	0.1450	9.54	0.2093	8.46
Methylation level	0.1666	6.79	0.3116	> 15.70	0.8127	> 15.70
GC content	0.2478	13.98	0.0257	0.57	-0.1815	5.84
Transcription level	-0.1744	7.31	-0.0294	0.69	0.0871	1.77
Female recombination rate	-0.0321	0.48	0.0533	1.60	0.0210	0.25
Male recombination rate	0.0971	2.49	0.0642	2.13	-0.0130	0.14
Explained variance	3.97%		1.73%		17.73%	
n^a^	5,550		14,165		3,376	

Dataset A contains no CpG islands (CGIs) or DNase I hypersensitive sites (DHSs)), dataset B contains DHSs but no CGIs, and dataset C contains both CGIs and DHSs. ^a^Sample size.

As a negative control we performed logit-regression analysis using CpH transition rate as the response variable, which we *a priori *did not expect to be affected by methylation level (Table 5). For all three datasets non-CpG divergence was the dominant explanatory variable (standardized slopes of 0.2559, 0.2205 and 0.2094), followed by GC content (-0.1366, -0.1389 and -0.1681), while DNA methylation level was only of marginal importance (all slopes < 0.003 and *P*-values > 0.01). Here, the percentage of explained deviance was in all cases > 50% (59.3, 66.3 and 73.2).

**Table 5:** Summary of parameter estimates and significance levels of the multivariate generalized linear regression analysis for CpH transition rate

	Dataset A		Dataset B		Dataset C	
			
	Estimate	-log_10_(p)	Estimate	-log_10_(p)	Estimate	-log_10_(p)
Divergence	0.2559	> 15.70	0.2205	> 15.70	0.2094	> 15.70
Methylation level	0	0	0.0036	1.34	0.0032	0.52
GC content	-0.1366	> 15.70	-0.1389	> 15.70	-0.1681	> 15.70
Transcription level	0.0084	3.08	0.0065	6.09	0.0063	1.59
Female recombination rate	0.0048	1.15	0.0002	0.04	0.0019	0.27
Male recombination rate	0.0083	2.71	0.0046	2.91	0.0014	0.19
Explained deviance	10,790		24,947		6,639	
Residual deviance	7,407		12,664		2,427	
n^a^	13,038		21,636		3,871	
AIC^b^	60,968		113,273		21,181	

Dataset A contains no CpG islands (CGIs) or DNase I hypersensitive sites (DHSs)), dataset B contains DHSs but no CGIs, and dataset C contains both CGIs and DHSs. ^a^Sample size. ^b^Akaike Information Criterion.

In order to examine if the, in most cases, relatively low percentage of explained deviance for CpG transition and transversion rates was a matter of stochastic noise on substitution rate estimates, we increased the sequence length of each data point by concatenating all introns of each gene from dataset A (*n *= 5,454 genes). We then performed logit-regression analysis following the above procedure (Table 6). There were no drastic changes in the pattern and importance of explanatory variables compared to the per-intron analysis of dataset A. As a slight change though, GC content was now the dominant explanatory variable for variation in CpG transition rate (-0.1229 standardized slope), closely followed by non-CpG divergence (0.1182) and then DNA methylation level (0.0413). With respect to the percentage of explained deviance, there was a general increase: 26.9% versus 17.4% for CpG transition rate, 8.4% versus 3.4% for CpG transversion rate, and 65.9% versus 59.3% for CpH transition rate.

**Table 6:** Summary of parameter estimates and significance levels of the multivariate generalized linear regression analysis for the set of concatenated introns

	CpG transition rate		CpG transversion rate		CpH transition rate	
			
	Estimate	-log_10_(p)	Estimate	-log_10_(p)	Estimate	-log_10_(p)
Divergence	0.1182	> 15.70	0.1549	> 15.70	0.1854	> 15.70
Methylation level	0.0413	10.21	0.0261	1.44	0.0035	0.61
GC content	-0.1229	> 15.70	-0.1404	> 15.70	-0.1423	> 15.70
Transcription level	-0.0150	2.44	-0.0320	2.82	0.0107	5.53
Female recombination rate	0.0059	0.61	0.0073	0.32	0.0056	1.72
Male recombination rate	-0.0047	0.44	-0.0066	0.29	0.0085	3.57
Explained deviance	880		333		6,538	
Residual deviance	2,390		3,614		3,387	
n^a^	5,454		5,454		5,454	
AIC^b^	21,263		14,384		30,905	

^a^Sample size. ^b^Akaike Information Criterion.

**Figure 1:**
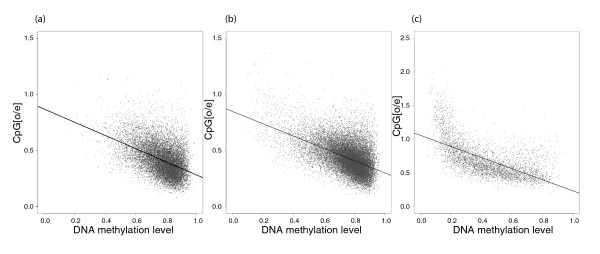
**Covariation between the observed versus expected CpG content (CpG_[o/e]_) and methylation level for intronic regions of the human genome**. The three panels show the covariation for datasets A, B and C, respectively (see text). Each grey dot represents one intronic region. The black dashed line is the linear regression line of a simple linear regression model.

**Figure 2:**
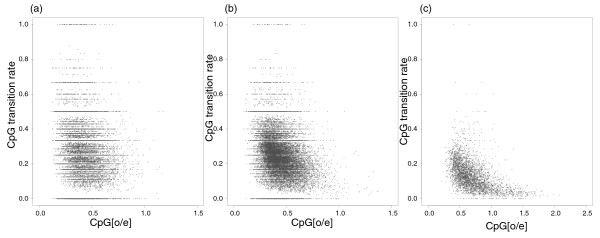
**Covariation between CpG transition rate and the observed versus expected CpG content (CpG_[o/e]_) for intronic regions of the human genome**. The three panels show the covariation for datasets A, B and C, respectively. Each grey dot represents one intronic region.

**Figure 3:**
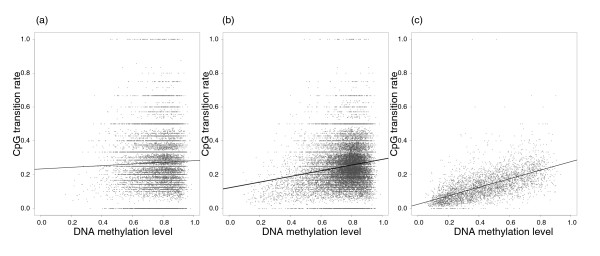
**Covariation between CpG transition rate and methylation level for intronic regions of the human genome**. The three panels show the covariation for datasets A, B and C, respectively. Each grey dot represents one intronic region. The black dashed line is the linear regression line of a simple linear regression model.

The ratio of the observed to the expected CpG content (CpG_[o/e]_) has been used as an indication of CpG mutability or a proxy for DNA methylation level. To qualitatively explore these relationships, we visualize the relationships between CpG_[o/e] _and DNA methylation level (Figure 1) and between CpG_[o/e] _and CpG transition rate (Figure 2). A high rate of CpG mutation should deplete the frequency of CpG sites so that CpG_[o/e] _decreases. Given the above demonstration that methylation level is positively correlated with CpG mutability, one would thus expect CpG_[o/e] _to be negatively correlated with methylation level. Figure 1 visualizes this negative correlation between CpG_[o/e] _and methylation level. Spearman rank correlation coefficients (*r_s_*) are -0.44, -0.47 and -0.67 for datasets A, B and C, respectively. As expected, CpG_[o/e] _and CpG transition rate are also negatively correlated (*r_s _*= -0.21, -0.30 and -0.58 for datasets A, B and C, respectively). As seen in Figure 2, this relationship might be nonlinear. Figure 3 shows the relationship between CpG transition rate and methylation level, the relationship that we explored quantitatively in the analyses presented above. With *r_s _*= 0.03, 0.14 and 0.67 for datasets A, B and C, respectively, note the difference in the strength of correlation between the sets of introns that do not contain CGIs (datasets A and B) and the set of introns that contains CGIs (dataset C).

**Figure 4:**
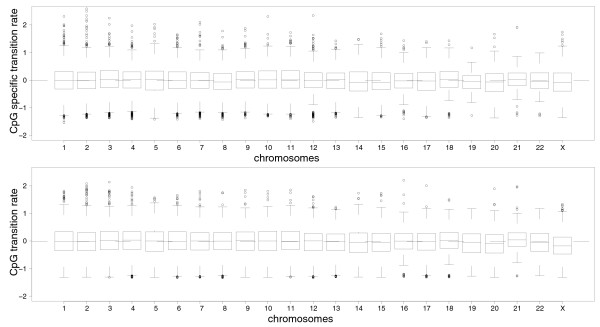
**Box plots of the CpG-specific transition rate (see Materials and methods) and CpG transition rate per chromosome**. The black dashed line represents the overall mean over all chromosomes.

Because of male-biased mutation, mutation rates are typically lower on the X chromosome than on autosomes [25,26] since the X chromosome spends approximately two-thirds of the time in females. We analyzed the sex-bias in CpG transition rate and CpG-specific transition rate (the CpG rate corrected for variation in non-CpG divergence; see Materials and methods) by comparing data from the X chromosome and the autosomes, where significance levels were based on a one-way ANOVA (Table 7). While CpG transition rate was generally higher on the autosomes than on the X chromosome, the CpG-specific transition rate was similar in the two chromosomal categories, indicating that methylation-induced mutations occur at similar frequencies on autosomes and the X chromosome. For visualization, box-plot diagrams are shown in Figure 4.

**Table 7:** Comparison of the CpG transition rate between the X chromosome and the 22 autosomes

	CpG specific transition rate		CpG transition rate	
		
Levels of the one-way ANOVA	Estimate	P-value	Estimate	P-value
Intercept	-0.0708	1.04e-03**	-0.1730	6.82e-15***
Chromosome 1	0.0638	1.58e-02*	0.1531	1.83e-08***
Chromosome 2	0.0565	3.32e-02*	0.1567	9.46e-09***
Chromosome 3	0.0870	1.21e-03**	0.1809	5.97e-11***
Chromosome 4	0.0644	2.46e-02*	0.1759	2.37e-09***
Chromosome 5	0.0437	1.20e-01	0.1374	2.03e-06***
Chromosome 6	0.0579	4.94e-02*	0.1666	3.86e-08***
Chromosome 7	0.0330	2.97e-01	0.1293	6.84e-05***
Chromosome 8	-0.0036	9.08e-01	0.1382	1.53e-05***
Chromosome 9	0.0942	2.71e-03**	0.1890	4.94e-09***
Chromosome 10	0.0871	2.95e-03**	0.1864	6.22e-10***
Chromosome 11	0.0988	1.44e-03**	0.1911	2.09e-09***
Chromosome 12	0.0555	6.11e-02	0.1345	1.03e-05***
Chromosome 13	0.0625	8.53e-02	0.1501	5.88e-05***
Chromosome 14	0.0005	8.88e-01	0.0900	9.89e-03**
Chromosome 15	0.0333	9.87e-01	0.1376	2.74e-05***
Chromosome 16	0.0630	1.13e-01	0.1707	2.92e-05***
Chromosome 17	-0.0011	9.74e-01	0.1144	1.33e-03**
Chromosome 18	0.0862	1.92e-02*	0.1901	5.02e-07***
Chromosome 19	-0.0118	8.68e-01	0.1157	1.15e-01
Chromosome 20	-0.0077	8.46e-01	0.0756	6.28e-02
Chromosome 21	0.1033	5.49e-02	0.2319	2.76e-05***
Chromosome 22	0.0288	6.00e-01	0.1228	2.99e-02*

CpG-specific transition rate is corrected for among-chromosomes variation in divergence, GC content, DNA methylation and transcription level; CpG transition rate is only corrected for variation in GC content, DNA methylation and transcription level among chromosomes. Estimates and *P*-values of a one-way ANOVA are listed, where the contrasts are based on the X chromosome. P-values are marked with asterisks to highlight their significance level, where single, double and triple asterisks indicate P-values below a threshold of 0.05, 0.01 and 0.001, respectively.

## Discussion

Our analysis shows that variation in the substitution rate of CpG sites in introns of human genes is mainly governed by three factors: non-CpG divergence, methylation level and GC content. Transcription level shows only a weak influence and recombination rate has no detectable importance. The relationship between CpG transition rate and non-CpG divergence, methylation level and GC content was similar in all three datasets defined by the presence or absence of intronic DHSs and/or CGIs: methylation level and divergence were consistently positively correlated to CpG transition rate whereas the local GC was always negatively correlated to CpG transition rate. This indicates that even if introns containing DHSs and/or CGIs are more likely to evolve under selective constraint, the overall pattern is not changed. Moreover, we note that non-CpG divergence is similar in the three datasets, suggesting that the impact of selection on intronic regions containing DHSs and/or CGIs as a whole is low. This does not exclude that selection acts on a subset of sites, but if so, the proportion of such sites within introns seems to be low. However, the relative importance of the three dominant explanatory variables differs between the three sets of introns; the presence or absence of CGIs especially impacts on the hierarchical level of explanatory variables. Non-CpG divergence and local GC content have a stronger impact than methylation level for introns not containing CGIs (datasets A and B), while for introns containing CGIs (dataset C) methylation level is the most important factor. If introns containing CGIs are under selection, it is difficult to envision a situation in which epigenetic marks (methylation level) affect the strength of selection at CpG sites. This is especially so if one considers that a positive correlation between methylation level and substitution rate would be obtained, either directly or indirectly. Rather, the fact that methylation level shows a much broader distribution in introns containing CGIs (Table 1, Figure 3), where low methylation levels are usually necessary for transcriptional activation, should provide higher power to detect correlative relationships including methylation level. This seems a more plausible explanation to the stronger correlation between methylation level and CpG transition rate in dataset C. In the following we discuss in further detail the three main variables that explain variation in the CpG substitution rate.

### The effect of non-CpG divergence

The significant correlation between CpG transition rate and non-CpG divergence demonstrates that regions more amenable to mutations in general are also more likely to show an increased CpG mutation rate. A significant proportion, likely the majority, of non-CpG mutations are thought to arise as the result of replication errors in mitotic cell divisions of the germ line [27,28]. Although most CpG mutations are considered to occur spontaneously, CpG sites should also be subject to replication errors where mutations are introduced independent of cytosine methylation status. If so, genomic parameters affecting the local rate of non-CpG mutation rate may contribute to variation in the CpG mutation rate as well. The correlation between CpG transversion rate and non-CpG divergence and the observation that the correlation between non-CpG divergence and CpG transition rate stays significant after incorporating methylation level support this explanation. Moreover, the finding that CpG transition rate is higher on autosomes than on the X chromosome when non-CpG divergence is not corrected for is in line with the predictions from male-biased mutation, which is considered a consequence of replication-induced mutations [25,26]. Note that a model of replication-associated CpG mutations may be reinforced by methylation level if methylated cytosines are more prone to replication errors than unmethylated cytosines.

Those factors that cause variation in the rate of non-CpG mutation may thus also cause variation in the rate of 5-methylcytosine to thymine substitution (for example, via the incidence of deamination or the efficiency of repair of 5-methylcytosine to thymine conversions). One such possibility is that ^m^CpG marks sequences for chromatin modification, or is the result of such modification, and that chromatin structure affects the overall mutation rate at non-CpG sites [12,29]. Another possibility is that both CpG and non-CpG substitution rates are related to replication timing in that the incidence of generalized DNA damage increases during replication [10].

### The effect of methylation level

Although certain types of sequences are known to be methylated at high (repeat elements, CGIs of inactivated genes) or low (CGIs of expressed genes) levels [30,31], methylation-induced CpG mutability has often been considered to occur genome-wide at a relatively constant rate. Recently, the correlation between GC content and CpG substitution rate has been interpreted as GC co-varying with methylation level [5], which would then be the cause of CpG mutation rate variation. Using multivariate regression analysis, our study provides evidence that both parameters independently impact on CpG mutation rate variation. However, while GC content affects cytosine mutability in general, intronic levels of methylation specifically affect the rate of CpG transitions; the analysis of variation in CpH transition rate and variation in the CpG transition/transversion rate ratio clearly shows that methylation level has the strongest effect on CpG transitions. In particular, the lack of correlation between methylation level and CpH transition rate argues against the possibility that methylation level co-varies with a parameter that has an overall effect on substitution rate variation. Chen *et al*. [11] found that both CpG and non-CpG substitution rate increases with replication timing. In the case of the non-CpG rate, this was suggested to result from an increased rate of damage, or decreased efficiency of repair, during replication. In the case of the CpG rate, they noted that methylation levels increase from early to late replicated regions so that the correlation between CpG rate and replication timing could be an indirect effect caused by the correlation between methylation status and replication timing. However, while not excluding an effect of replication timing on the CpG rate, our results demonstrate that the link between CpG and non-CpG rates is independent of methylation status. A difference between our study and that of Chen *et al*. [11] is that they incorporated methylation data from approximately 2,000 PCR amplicons on three different human chromosomes while we utilized whole-genome methylation data from a total of more than 38,000 introns each more than1 kb in length.

An observation that further testifies to the role of methylation level on the rate of CpG transition is the correlation between methylation level and the CpG transition/transversion rate ratio (κ). For dataset C, where the methylation level was of the broadest range, methylation was by far the most important factor explaining the variation in κ (standardized slope of 0.81). Our results thus point out the impact of methylation level specifically on CpG transition rate variation. Moreover, several arguments suggest that the effect of methylation level on CpG transition rate that we observe is, in fact, underestimated. First, data on methylation levels were from mature sperm cells, whereas most mutations in the male germ line are expected to occur during mitosis in spermatogonia. Although methylation levels of sperm cells and spermatogonia are correlated [32], they may not be identical. Second, data on methylation levels represent the present day state of epigenetic modification, whereas the estimate of CpG substitution rate is averaged over millions of years of human evolution. If methylation patterns have changed over this evolutionary time scale, we should expect the correlation between methylation level and substitution rate to be reduced. Third, since the sex-bias in the rate of CpG mutation is small [2,33,34], maternally originating CpG mutations should contribute significantly to the CpG substitution rate. Since data on methylation levels from human oogenesis are lacking, we are unable to include female methylation level as an explanatory variable. Given that methylation patterns may very well differ between the male and female germ line, as it does between sperm cells and somatic tissues [31], using only methylation data from the male germ line should reduce the observed correlation between methylation level and CpG substitution rate.

### The effect of GC content

We found a negative correlation between CpG substitution rate and local GC content, confirming similar results from previous work [5,14,15,35] (but see [36]). It has been argued that high GC content stabilizes double-stranded DNA and thereby reduces mutation rate [5,15,35]. Deamination requires physical separation of the two DNA strands. The thermodynamic stability of the double-stranded DNA should be influenced by the local GC content since G:C bonds are stronger than A:T bonds. This makes intuitive sense. However, the negative correlation with GC content is not specific to CpG substitution rate since we also find a significant negative correlation between CpH transition rate (that is, the C to T transition rate in non-CpG sites) and GC content. Correlations to GC content need careful interpretation as GC content itself co-varies with many other genomic parameters. More work will clearly be needed here to, for example, test hypotheses on the role of biased gene conversion [35] or on regional variation in the rate of deamination of unmethylated cytosines [15] to explain the correlation between non-CpG divergence and GC content.

### The effect of germ line transcription level

Although the effect was generally very low (standardized slopes < 0.015 for CpG transitions and 0.03 to 0.04 for CpG transversions), there were consistently negative correlations between CpG substitution rate and transcription level. In contrast, the C to T transition rate at non-CpG sites (that is, the CpH transition rate) was positively correlated with transcription level. Even though the effect was weak, the direction of correlation was coherent among all three sets of introns analyzed. It has previously been shown that C to T transitions in non-CpG sites can be induced during active transcription, known as transcription-induced mutations [9,37,38], which is reflected by a strand bias in mutation rate. As we find no evidence for strand bias in CpG substitution rate, it seems unlikely that the negative correlation between transcription level and CpG substitution rate reflects an influence via a strand-specific mechanism, such as transcription-induced mutations, or the efficiency of transcription-coupled repair, a repair mechanism that repairs bulky lesions during active transcription [39-41]. It might, however, reflect higher accessibility to DNA for repair enzymes specific to methylated CpG sites during active transcription [12,29].

## Conclusions

Our study found a significant correlation between the extent of germ line methylation and the substitution rate at human CpG sites. It thus provides novel and direct empirical support for a link between epigenetic imprinting and the rate of molecular evolution. We also show that the CpG substitution rate is positively correlated with non-CpG divergence, suggesting common factors involved in governing overall mutation rate variation in the human genome. These results will help in understanding the causes of mutation rate variation and will thus help in formulating neutral models of sequence evolution.

## Materials and methods

### Sequence data

Alignments of orthologous intronic regions for human (GRCh37), rhesus macaque (MMUL1.0) and mouse (NCBIM37) were retrieved as part of the 11-way eutherian mammal whole-genome alignments from the Ensembl database release 59 via the Ensembl perl Application Programme Interfaces (APIs). Positions of intronic regions were established based on human exonic regions extracted from Known Genes dataset at the UCSC table browser [42], where intronic regions (and their aligned sequences) were defined as all non-exonic regions within a human gene. We restricted our dataset to introns that fell within a single synthenic alignment block. To reduce stochasticity in downstream analysis, we further limited our dataset to introns with a minimum length of 1,000 unambiguous sites, of which there were 56,363.

### Estimation of dinucleotide substitution rates

For estimation of three types of dinucleotide substitution rate - CpG transition rate, CpG transversion rate and CpH transition rate (where H is either A, C or T) - we used the PAML software package version 4.1 [43]. We applied the general time-reversible substitution model implemented in baseml and allowed no substitution rate variation within an intron. For estimation of dinucleotide substitution rates we first computed the marginal probability distribution of intronic sequences of the common ancestor of human and rhesus macaque using mouse as an outgroup. As parsimonious sequence reconstruction is critical when it comes to the assignment of the ancestral CpG state, we used maximum-likelihood-based ancestral sequence reconstruction [44]. Then we estimated human-specific dinucleotide substitution rates based on the parsimony principle by comparison of the reconstructed ancestral sequences and the human sequences. Here, CpG transition rate was defined as the number of CpG to TpG mutations per CpG site occurring on either of the two DNA strands (that is, CpG to TpG or CpG to CpA). Only in order to address the mutational strand bias in CpG mutation rate, we distinguished between two cases: CpG to TpG occurring on the coding strand or CpG to TpG occurring on the non-coding strand. Analogously, CpG transversion rate was defined as the number of CpG to RpG mutations (where R is either A or G) per CpG site occurring on either of the two DNA strands, and CpH transition rate was defined as the number of CpH to TpH mutations per CpH site occurring on either of the two DNA strands.

Given the evolutionary distance between human and rhesus macaque, we cannot exclude the possibility that more than a single mutation has occurred at CpG sites, which our method based on the parsimony principle assumes by necessity. This may lead to CpG rates being underestimated. To address how this could potentially affect the downstream statistical analyses of the influence of different explanatory variables on CpG rates, we compared human-specific CpG transition rates estimated using mouse-rhesus macaque-human alignments and rhesus macaque-chimpanzee-human alignments. Further, we compared CpG transition rates estimated with the method described here with a pure counting method. As described in Additional file 1, while we are likely to underestimate the CpG transition rate in the human-rhesus monkey comparison, we are still able to capture the general tendency of high versus low CpG-diverged regions. Since our downstream statistical analyses aim to explain the variation in CpG transition rate, and not to quantify the CpG transition rate, inferences based on our results should not be critically affected.

### Estimation of nucleotide divergence

We estimated human-specific non-CpG divergence for the set of 56,363 introns as the divergence between human and the ancestor of human and rhesus macaque after all sites showing a CpG in any of human, rhesus macaque and mouse had been masked. Again, estimation was based on the PAML software package version 4.1 and the general time-reversible substitution model implemented in baseml allowing no substitution rate variation within an intron.

### DNA methylation level

We downloaded data on whole-genome human sperm cell DNA methylation levels from Array-Express experiment E-TABM-482 [22]. Methylation levels - that is, the fraction of methylated CpG sites among all CpG sites - were calculated for 100-bp windows throughout the genome. We extracted those 100-bp windows located within introns to compute average DNA methylation levels of introns weighted by the number of CpG sites per window. From the initial set of 56,363 introns we discarded introns containing less than five windows for which methylation status was available. It should be noted that we used methylation levels in human spermatozoa as an indicator of germ line methylation status. In theory, methylation levels might differ between spermatozoa and germ cells; however, it has recently been shown that sperm cell and germ cell DNA methylation status are highly correlated, at least in mice [32].

### Gene expression data set

We used human Affymetrix exon array expression data from testis [45], evaluated by Xing *et al*. using a probe selection algorithm [46], to determine the level of transcriptional activity in germ cells. Three repeated measurements of gene expression values, denoted as expression indices, were available. Following Xing *et al*. [45], we took the logarithm of the expression indices to gain a measure approximately linearly proportional to transcription levels in germ cells. Subsequently, mean values were computed for each set of repeated measurements. Assignments of expression values to Ensembl gene IDs were based on a table extracted from the UCSC table browser. Finally, we assigned each intronic region the transcription level of its respective gene.

### Recombination rate

We used deCODE female and male human recombination rate estimates for 1-Mb windows reported by Kong *et al*. [47]. The coordinates for the 1-Mb windows are based on the NCBI36 assembly of the human genome. As estimates of substitution rates used in our study were based on the GRCh37 assembly, we projected the 1-Mb windows onto the latter assembly via the coordinate translation utility of the Ensembl database.

### DNA sequence composition

Intronic GC content was defined as the frequency of guanine and cytosine in all unambiguously assigned sites. The ratio of observed versus expected CpG content (CpG_[o/e]_) was computed as the frequency of CpG sites divided by the frequency of C and G:

where p(X) represents the frequency of X in the number of unambiguously assigned sites of the respective intron.

### Classification of introns

There is clear evidence of functional constraints in non-coding DNA (reviewed in [48]), which is likely to include intronic sequences. This might affect attempts to find correlations between the intronic CpG substitution rate and other genomic parameters since selection, if strong enough, could blur existing correlations. In contrast, it is unlikely that selection adds to existing correlations, unless there is interaction between the strength of selection and the genomic parameters under consideration. To investigate the potential impact of selection, we considered two types of potential regulatory sequences contained within introns, CGIs and DHSs. CGIs, which are often hypomethylated in many tissues, tend to be involved in transcription regulation and hence might be under selection. By the same token, DHSs, which are sites easily accessible to DNA proteins and indicators of open chromatin structure, are commonly considered to identify the location of potential regulatory elements [49]. CGIs were identified based on the Gardiner-Garden criteria [50] and DHSs were identified as part of the ENCODE pilot project [51], the latter available through the Ensembl Human Regulatory build version 8. Note that the Gardiner-Garden criteria are only sequence-based and may thus not be specific for *bona fide *CGIs [52]. We then classified introns on the basis of the presence or absence of such sequences. Dataset A was defined as introns containing neither CGIs nor DHSs (13,038 introns), dataset B was defined as introns containing DHSs but not CGIs (21,636), while dataset C was defined as introns containing both CGIs and DHSs (3,871). Dataset A might represent introns most closely evolving neutrally. We discarded 41 introns containing CGIs but no DHSs from the analysis.

### Multivariate generalized linear regression analysis

All statistical analyses were performed with the software package R version 2.7.2. We performed generalized linear regression analysis for a set of 38,586 introns where estimates for each of the three dinucleotide substitution rates as response variables and estimates of non-CpG divergence, DNA methylation level, intronic GC content, germ line transcription level and DNA recombination rate as possible explanatory variables were available. We implemented a logit link function and binomially distributed error terms in the generalized linear model, as estimates of dinucleotide substitution rates were binomial data. To reduce skewness in the distribution of recombination rate, recombination rate was log-transformed to base 10, after adding a constant of 0.01 in order to allow for zero rate values. Regression analysis was performed after Z-transformation of the explanatory variables, which means standardization of the mean value to 0 and of the standard deviation to 1. Note that the term 'explanatory variable' is meant in a statistical context and does not necessarily imply a causative role of a particular variable.

### Sex-bias in mutation rate

To evaluate the strength of sex-bias in methylation-induced CpG transition rate, we compared CpG transition rate of autosomal and X-linked introns by applying a one-way ANOVA to CpG transition rates grouped by chromosomes with the contrast matrix based on the X chromosome. We corrected CpG transition rates for variation in non-CpG divergence, methylation level, intronic GC content, germ line transcription level and recombination rate among chromosomes, which we denoted as CpG-specific transition rate. For comparison, one-way ANOVA was also performed without correction for variation in non-CpG divergence among chromosomes, simply denoted as CpG transition rate.

## Abbreviations

bp: base pair; CpG_[o/e]_: observed versus expected CpG content; CGI: CpG island; DHS: DNase I hypersensitive site; κ: CpG transition/transversion rate ratio; ^m^CpG: methylated CpG.

## Competing interests

The authors declare that they have no competing interests.

## Authors' contributions

CFM did the analyses and drafted the manuscript. HE conceived of the study and helped to draft the manuscript.

## Supplementary Material

Additional file 1 **Supplementary text and Supplementary figures S1 and S2**. Click here for file
