# Supplementary material for: Substitution rate variation at human CpG sites correlates with non-CpG divergence, methylation level and GC content
Source: Genome Biol. 2011 Jun 22;12(6):R58. doi: 10.1186/gb-2011-12-6-r58 (PMC3218846; doi:10.1186/gb-2011-12-6-r58)
Supplement: Additional file 1 — Supplementary text and Supplementary figures S1 and S2. [file gb-2011-12-6-r58-S1.DOC]

# Supplementary Material

**Substitution rate variation at human CpG sites correlates with non-CpG divergence, methylation level and GC content**

**Carina Farah Mugal and Hans Ellegren^1^**

We estimated the three types of dinucleotide substitution rates in the human lineage, CpG transition rate, CpG transversion rate and CpH transition rate, based on a comparison of human intronic sequences to their marginal probability distribution of reconstructed human and rhesus macaque ancestral intronic sequences. Two critical aspects in this estimation procedure are the assumptions of site independency and that differences between ancestral and human dinucleotides are a result of a single nucleotide substitution, i.e. that each dinucleotide experienced at maximum one nucleotide substitution.

The assumption that each dinucleotide experienced at maximum one nucleotide substitution is problematic if species are far diverged. This is especially so for CpG transitions which are known to occur about 10 times more frequently than other substitutions in the human genome. Our estimation procedure will miss CpG transitions that were followed by any other nucleotide substitution or that occurred subsequent to a mutation which resulted in a CpG dinucleotide. To investigate the effect of such cases of multiple substitutions on substitution rate estimates, and on subsequent downstream analyses on the relationship between substitution rate variation and explanatory variables, we compared our original human-specific CpG transition rate estimates based on human and rhesus macaque ancestral sequences (HM estimates, with mouse used as outgroup) to estimates based on human and chimpanzee (CHIMP2.1) ancestral sequences (HC estimates). In the latter case we used rhesus macaque as an outgroup. As human and chimpanzee sequences are on average less than 1.5% diverged, the assumption that each dinucleotide experienced at maximum one nucleotide substitution is less of a concern. However, stochastic variation is likely to have a higher impact on substitution rate estimates in this case, especially if the number of CpG dinucleotides per intron is low (which is essentially why we did not use the rhesus macaque-chimpanzee-human trio for our main analyses). We then investigated the correlation between HC and HM estimates per intron as a function of the minimum number of CpG dinucleotides per intron (Supplementary figure 1A). The correlation is moderate (0.5) for introns that contain at least 20 ancestral CpG sites but increases to >0.9 for introns that contain at least 500 CpG sites. For introns with at least 100 CpG sites we visualize the relationship between the number of ancestral CpG sites per intron in the HC and HM data in Supplementary figure 1B) and the relationship between HC and HM CpG transition rate estimates in Supplementary figure 1C. While it seems clear that we underestimate the number of ancestral human and rhesus macaque CpG sites, this does not seem to affect the linear relationship between HC- and HM-derived CpG transition rate estimates. Thus, any downstream statistical analysis that aims to explain variation in CpG transition rate is not likely to be biased by our approach for estimation of the CpG transition rate in the rhesus macaque-human comparison.

One possible reason why we underestimate the number of ancestral human and rhesus macaque CpG sites might be the assumption of site independency. As CpG transitions occur frequently, the likelihood of convergent CpG transitions in the human and rhesus macaque lineage is higher than convergent evolution of other types of nucleotide substitutions. This means that the likelihood of convergent CpG transitions is higher than expected under a single site nucleotide substitution model. Thus, the probability of CpG sites mutating in both lineages is likely to be underestimated in the marginal probability distribution, which is based on a single nucleotide substitution model. We investigated this issue further by counting the number of events where the mouse sequence shows a CpG and both the human and the rhesus macaque sequences showed either a TpG or a CpA dinucleotide (some of which may represent independent CpG transitions in the terminal primate branches rather than a single CpG transition in the lineage leading to the common ancestor of rhesus macaque and human or a TpG to CpG, or CpA to CpG, transition in the rodent lineage). This number was added to the number of events where mouse and rhesus macaque sequences show a CpG site while the human intronic sequence shows either a TpG or a CpA dinucleotide, which leads to an alternative CpG transition rate estimate. We call this method to estimate the CpG transition rate “Count method”, whereas the original method is in this context referred to as “ML method”. In Supplementary figure 2 we visualize the relationship between CpG transition rate estimates (panel A), the number of ancestral CpG sites (panel B) and the number of CpG transitions (panel C) estimated using the Count method and the ML method for the set of introns that contain at least 100 CpG sites. This shows that estimates of both the number of ancestral CpG sitess and the number of CpG transitions are higher using the Count method than using ML method. Moreover, the comparison of rate estimates shows that we are likely to underestimate the CpG transition rate using the ML method, especially in regions where the CpG transition rate is high. However, again the relationship between rate estimates appears rather linear. Any downstream statistical analysis that aims to explain variation in CpG transition rate may possibly face a reduction in power but the general pattern should not be affected. Hence, we conclude that even though the absolute values of the CpG transition rate estimates are likely to be underestimated and that our estimation procedure may lead to a reduced signal/noise ratio, the general patterns seen in the downstream statistical analysis should not be critically affected. A higher resolution in the rate estimation procedure may however lead to better resolution in exploring the relationship between CpG transition rate and its possible explanatory variables.

**Figure legends**

Supplementary Figure 1: Correlation between CpG transition rate estimates. Panel A shows the correlation of HC and HM estimates as a function of the minimum number of CpG sites per intron. The red circle indicates the correlation for the set of introns that contain at least 100 CpG sites per intron. For this set of introns the number of ancestral human and chimp CpG sites versus ancestral human and macaque CpG sites are shown in panel B. Here, one dot represents one intron and the dashed line indicates a 1:1 linear relationship. In panel C CpG transition rate estimates for the same set of introns are plotted against each other. Again, one dot represents one intron and the dashed line represents a type II linear regression line.

Supplementary Figure 2: “Count method” versus “ML method”. Panel A shows the relationship between CpG transition rate estimates for the set of introns that contain at least 100 CpG sites. Panel B shows the relationship between the number of ancestral CpG sites for the same set of introns. Panel C shows the relationship between the number of CpG transitions for the same set of introns. In all three panels one dot represents one intron and the dashed line indicates a 1:1 linear relationship.

Supplementary figure 1

Supplementary figure 2
